# Antarctic Soils Select Copiotroph-Dominated Bacteria

**DOI:** 10.3390/microorganisms12081689

**Published:** 2024-08-16

**Authors:** Lujie Zhang, Xue Zhao, Jieying Wang, Liyuan He, Chengjie Ren, Jun Wang, Yaoxin Guo, Ninglian Wang, Fazhu Zhao

**Affiliations:** 1 Shaanxi Key Laboratory of Earth Surface System and Environmental Carrying Capacity, Northwest University, Xi’an 710127, China; 2 College of Urban and Environmental Sciences, Northwest University, Xi’an 710127, China; 3 Oak Ridge National Laboratory, Environmental Sciences Division and Climate Change Science Institute, Oak Ridge, TN 37831, USA; 4 College of Agronomy, Northwest A&F University, Yangling 712100, China; 5 State Key Laboratory of Soil Erosion and Dryland Farming on the Loess Plateau, Institute of Soil and Water Conservation, Chinese Academy of Sciences and Ministry of Water Resources, Yangling 712100, China; 6 Shaanxi Key Laboratory for Carbon Neutral Technology, Northwest University, Xi’an 710127, China; 7 The College of Life Sciences, Northwest University, Xi’an 710072, China

**Keywords:** soil bacterial community, life strategies, Antarctic soil, forest soil, soil resource conditions

## Abstract

The life strategies of bacterial communities determine their structure and function and are an important driver of biogeochemical cycling. However, the variations in these strategies under different soil resource conditions remain largely unknown. We explored the bacterial life strategies and changes in structure and functions between Antarctic soils and forest (temperate, subtropical, and tropical) soils. The results showed that the weighted mean rRNA operon copy number in temperate soils was 19.5% lower than that in Antarctic soils, whereas no significant differences were observed among Antarctic, subtropical, and tropical soils. An unexpected result was that bacterial communities in Antarctic soils tended to be copiotrophs, such as Actinobacteriota and Bacteroidota, whereas those in temperate soils tended to be oligotrophs, such as Acidobacteriota and Chloroflexi. Functional predictions showed that in comparison to copiotrophs in Antarctic soils, temperate-inhabiting oligotrophic bacteria exhibited an 84.2–91.1% lower abundance of labile C decomposition genes (hemicellulose, cellulose, monosaccharides, and disaccharides), whereas a 74.4% higher abundance of stable C decomposition (lignin). Genes involved in N cycling (nitrogen fixation, assimilatory nitrate reduction, and denitrification) were 24.3–64.4% lower in temperate soils than in Antarctic soils. Collectively, our study provides a framework for describing the life strategies of soil bacteria, which are crucial to global biogeochemical cycles.

## 1. Introduction

The selection of life strategies in bacterial communities plays an important role in defining soil structure and regulating biogeochemical cycles [1]. However, the mechanisms by which bacterial life strategies interact with soil resource conditions remain controversial. Previous studies have found increases in copiotrophs (r-strategists) but decreases in oligotrophs (K-strategists) with increasing resource availability [2,3,4]. However, Peng et al. [5] and Yang et al. [6] reported contrasting findings. These inconsistent results demonstrate that bacteria vary in their life strategy selections owing to highly complex and heterogeneous soil conditions. Further understanding of the generalizable patterns of bacterial life strategies under different soil conditions can help mechanistically link microbial processes with biogeochemical cycles. 

The weighted mean rRNA operon copy number (*rrn*) has been used to identify bacteria life strategies [4,7,8]. Copiotrophs with higher *rrn* copies tended to have higher maximal growth rates and respond more quickly to resources (compared to oligotrophs) [9,10]. Based on 16S rRNA gene amplicon sequencing, Bacteroidetes and Acidobacteriota are often characterized as representative copiotrophic and oligotrophic bacteria, respectively [11,12]. Nevertheless, bacterial taxa belonging to a phylum may vary in resource preferences or functions, and it is unlikely for an entire phylum to display collective life strategies [13,14,15]. For example, in the phylum Proteobacteria, the classes γ-proteobacteria are often characterized as oligotrophs [12], while β-proteobacteria are classified as copiotrophs [16]. Therefore, the weighted mean rRNA operon copy number provided a community-level microbial trait [9,17]. However, there is limited information on the combination of community-level traits and finer phylogenetic and taxonomic resolutions to understand the response of bacterial life strategies under natural soil conditions, particularly harsh soil conditions.

Copiotrophic and oligotrophic bacteria differ in their functional genes associated with C cycling and N cycling [14,18]. These functions provide critical insights into the relationship between life strategies and biogeochemical cycling from the perspective of microbial genes. For instance, copiotrophs have a higher abundance of labile C decomposition genes, whereas oligotrophs have a higher abundance of stable C decomposition genes [14,19]. However, studies on the impact of life strategies on bacterial functional potential, especially N cycling functional potential, are still scarce. Exploring the differences in the datasets of functional genes is crucial for the comprehensive evaluation of oligotrophic and copiotrophic bacteria.

To fill this knowledge gap, we chose Antarctic soils with lower resource conditions and forest (temperate, subtropical, and tropical) soils with higher resource conditions. Soil nutrients (SOC, NH_4_^+^-N, NO_3_-N, and MBC) and the bacterial characteristics (weighted mean rRNA operon copy number, structure, and functional potential) were measured. We hypothesized that *rrn* copies in forest soils would be higher than those in Antarctic soils. In other words, the bacterial life strategies of Antarctic soils tend to favor oligotrophic taxa, whereas those of forest soils are dominated by copiotrophic taxa. Because soil nutrients were significantly lower in Antarctic soils than in forest soils [20,21] (Table 1). Therefore, the objectives of this study were to (i) determine *rrn* copies of bacteria and their life strategies in Antarctic and forest (temperate, subtropical, and tropical) soils and (ii) investigate bacterial community structure and functional potentials in different soils.

## 2. Materials and Methods

### 2.1. Study Area

This study was conducted in forest and Antarctic ecosystems (Figure S1). Ten forests within 3425 km of China were selected, covering temperate, subtropical, and tropical forest ecosystems (Figure S1; Table S1), which are well-protected national nature reserves. Temperate forest ecosystems include Maoer Mountain (ME), Dongling Mountain (DL), Fuxian (FX), and Huoditang (HDT). Subtropical forest ecosystems include Maoxian (MX), Gongga Mountain (GG), Mulun Station (ML), and Ailao Mountain (AL). Tropical forest ecosystems include Xishuangbanna (XSBN) and Jianfengling (JFL). Mean annual precipitation (MAP) and mean annual temperature (MAT) of these forests ranged from 486 to 2266 mm and 3.1 to 23.2 °C, respectively. 

Antarctic soil samples were collected from Inexpressible Island (approximately 50 km^2^), North Victoria Land, in the Ross Sea region (Figure S1). The interannual variation of temperature is significant in Inexpressible Island, with monthly average temperature below 0 °C and MAT ranging from −18.7 to −15.3 °C [22]. In addition, the island experiences low precipitation and a dry climate [23]. Detailed information on the forest and Antarctic soils is provided in Table S1.

### 2.2. Soil Sampling

Forest soil samples were collected from July to August 2019. At each selected forest, six plots (50 m × 50 m) were used with similar geographical characteristics as six independent replicates. Nine topsoil samples (0–10 cm) of each plot were collected using a 5 cm diameter soil auger along an “S” shape and combined into a composite sample. In total, 60 soil samples were collected from 10 sites.

The Antarctic soil samples were collected between December 2018 and February 2019. On the twelve selected plots (50 m × 50 m), nine topsoil samples (0–10 cm) of each plot were collected using a 5 cm diameter soil auger along an “S” shape and combined into a composite sample. Then, to ensure that the Antarctic soil samples are representative, the four samples were combined into a replicate based on the geographical characteristics. Finally, three independent Antarctic soil samples were obtained.

Fresh soil from the forest and Antarctica was sieved through a 2 mm mesh, and visible roots and residues were removed. After sieving, the soil (2–3 kg) was divided into two subsamples. One portion (approximately 60 g) was used for analyzing soil nutrient contents, and another portion was stored at −20 °C for DNA extraction.

### 2.3. Soil Physicochemical Properties Analyses

Soil pH (soil: water = 1:2.5) was measured using a pH meter (PHS-3C, Shanghai, China) [24]. Soil organic carbon (SOC) was measured using the K_2_Cr_2_O_7_ oxidation method. Soil NH_4_^+^-N and NO_3_^−^-N were estimated using KCl digestion. The soil microbial biomass C (MBC) of fresh soil samples was determined using the chloroform fumigation-extraction techniques described by Wang et al. [25].

The results indicated that soil nutrients were significantly lower in Antarctic soils than in forest soils (Table 1). Specifically, SOC, NH_4_^+^-N, and NO_3_^−^-N in forest soils were 1827–824%, 650–523%, and 868–509% significantly higher than in Antarctic soils, respectively (*p* < 0.05). The MBC of temperate soils was significantly higher than that of Antarctic soils by 434% (*p* < 0.05). In addition, MBC increased by 179% and 160% in subtropical and tropical soils, respectively, compared to Antarctic soils (*p* > 0.05).

### 2.4. DNA Extraction, PCR Amplification and Data Processing

Six replicates were conducted for each forest soil sample and three replicates were conducted for each Antarctic soil sample. Soil DNA was extracted from fresh forest and Antarctic soil samples using the E.Z.N.A.® soil DNA kit (Omega Bio-tek, Norcross, GA, USA), following the manufacturer’s instructions. Bacterial characteristics were determined using the 16S rRNA gene to amplify the V3–V4 region sequences and PICRUSt-estimated metagenomes. Taxonomy was assigned to Operational Taxonomic Units (OTUs) using the classify-sklearn naïve Bayes taxonomy classifier in the feature-classifier plugin with a 97% sequence similarity level. Further descriptions of DNA extraction, DNA quality and concentration, PCR amplification, and data processing by PICRUSt2 methods are provided in the Supplementary Materials. All the sequencing data have been deposited on the National Center for Biotechnology Information website (SRP504188).

According to the closest relatives with known rRNA operon copy numbers for each OTU, the rRNA operon copy numbers were estimated through the rrnDB database [26]. The mean operon copy numbers of the immediate child taxa were assigned to each OTU. If the mean copy number was unavailable, the mean copy number of its parent was used. For each sample, the weighted mean rRNA operon copy number was computed by multiplying the estimated operon copy number by the relative abundance of each OTU, which was then summed across all OTUs in the sample.

This study predicted the profiles of bacterial functional potentials related to C cycling and N cycling. Generally, the functional potential of C-decomposition genes includes labile C composition (monosaccharides, disaccharides, polysaccharides, hemicellulose, cellulose, and aminosugar) and stable C composition (lipids, chitin, and lignin) [27,28]. The functional potential of N cycling includes assimilatory nitrate reduction, dissimilatory nitrate reduction, denitrification, nitrogen fixation, and nitrification [29]. Microbial functions of C cycling and N cycling were predicted by PICRUSt2 upon KEGG databases.

### 2.5. Statistical Analysis

All analyses were performed using R v4.2.2 (RCoreTeam, Vienna, Austria). Alpha diversity index (Shannon index and the observed richness index) were calculated with the ‘picante’ package. The beta diversity was calculated by the ‘vegan’ package. Spearman correlations between soil nutrients and bacterial community at the phylum level and between *rrn* copies and environmental conditions (pH, MAP, and MAT), soil nutrients, and bacterial functional potential were performed using the “Hmisc” package. Linear discriminant analysis effect size (LEfSe) was used to detect differentially abundant taxa among four ecosystems by the ‘microeco’ package (LDA score > 3, *p* < 0.05). One-way analysis of variance (ANOVA) was performed to assess the effects of differences among four ecosystems on soil nutrients (SOC, NH_4_^+^-N, NO_3_^−^-N, MBC, and C/N), alpha diversity index, beta diversity, the weighted mean rRNA operon copy number, and potential function (C cycling and N cycling). All graphs were conducted using the R package “ggplot2” and further composed with Adobe Illustrator 2020 (Adobe Systems Incorporated, San Jose, CA, USA).

## 3. Results

### 3.1. Life Strategies in Antarctic and Forest Soils 

**Figure 1:**
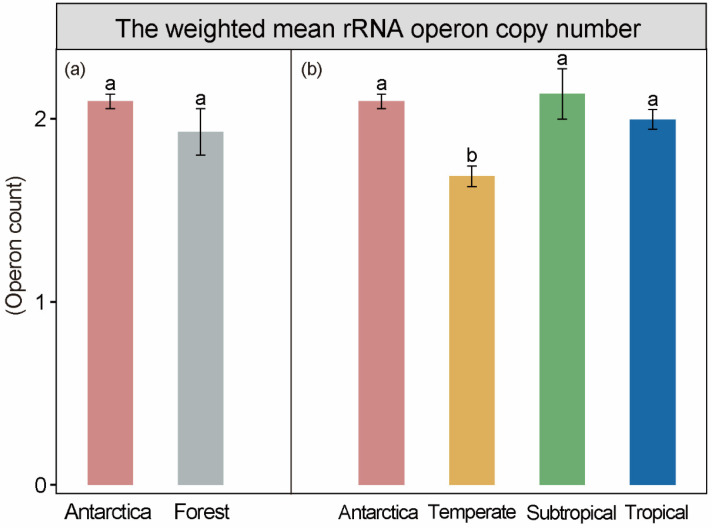
Life strategies of bacterial communities for studied sites. (**a**) Difference in the weighted mean rRNA operon copy numbers between Antarctica and forest soils; (**b**) difference in the weighted mean rRNA operon copy numbers among four ecosystems (mean ± SE). Different lowercase letters represent significant differences at 0.05 level.

We calculated the weighted mean rRNA operon (*rrn*) copy number to shed light on the bacterial community-level traits. Our results showed that *rrn* copies were 8.0% lower in forest soils than in Antarctic soils (*p* > 0.05; Figure 1a). Specifically, *rrn* copies in temperate soils were the lowest among the four ecosystems and were significantly lower than those in Antarctic soils by 19.54% (*p* < 0.05; Figure 1b). Furthermore, *rrn* copy numbers in subtropical and tropical soils were not significantly different from those in Antarctic soils (*p* > 0.05). Specifically, *rrn* copy numbers in subtropical soils were 1.91% higher than those in Antarctic soils, whereas they were 4.74% lower in tropical soils than in Antarctic soils (Figure 1b). The results indicated that the bacterial communities in Antarctic, subtropical, and tropical soils were dominated by copiotrophs (r-strategists), whereas those in temperate soils were dominated by oligotrophs (K-strategists).

### 3.2. Bacterial Community Diversity and Composition in Antarctic and Forest Soils

**Figure 2:**
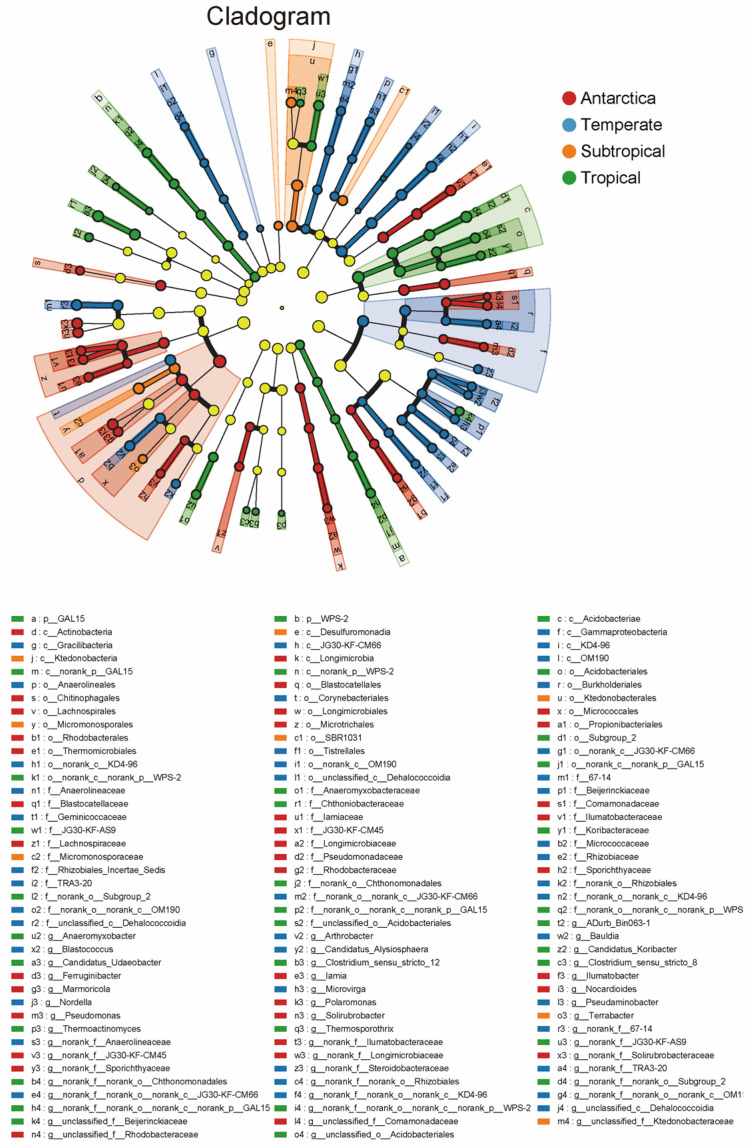
Linear discriminant analysis effect size (LEfSe) showed the significantly different taxa of bacterial communities for studied sites. The taxa with significantly different abundances among four ecosystems are represented by colored dots. The cladogram consists of six rings, representing the domain (innermost), phylum, class, order, family, and genus (outermost), respectively. The taxa with the LDA > 3.0 and *p* < 0.05 are shown.

Our results showed that the bacterial Shannon index and richness index were significantly lower in Antarctic soils than in temperate, subtropical, and tropical forest soils (*p* < 0.05; Figure S2a,b). The Shannon index and richness index in forest soils were 10.05–23.29% and 85.07–146.87% higher than those in Antarctic soils, respectively (Figure S2a and b). Moreover, using non-metric multidimensional scaling analysis (NMDS), we found that the bacterial communities were significantly different among the four ecosystems (Figure S2c). Further analysis using the LEfSe method showed that the bacteria enriched in Antarctic soils mainly belonged to Actinobacteriota, Bacteroidota, and Gemmatimonadota (Figure 2). The bacteria enriched in temperate soils mainly belonged to Proteobacteria (especially Gammaproteobacteria), Chloroflexi, Planctomycetota, and Patescibacteria (Figure 2). The bacteria that were mainly distributed in subtropical soils belonged to Desulfobacterota, Chloroflexi, and Actinobacteriota (Figure 2). The bacteria mainly distributed in tropical soils were GAL15, WPS-2, Armatimonadota, Verrucomicrobiota, Myxococcota, and Acidobacteriota (Figure 2).

### 3.3. Bacterial Functional Potential of C and N Cycling in Antarctic and Forest Soils

**Figure 3:**
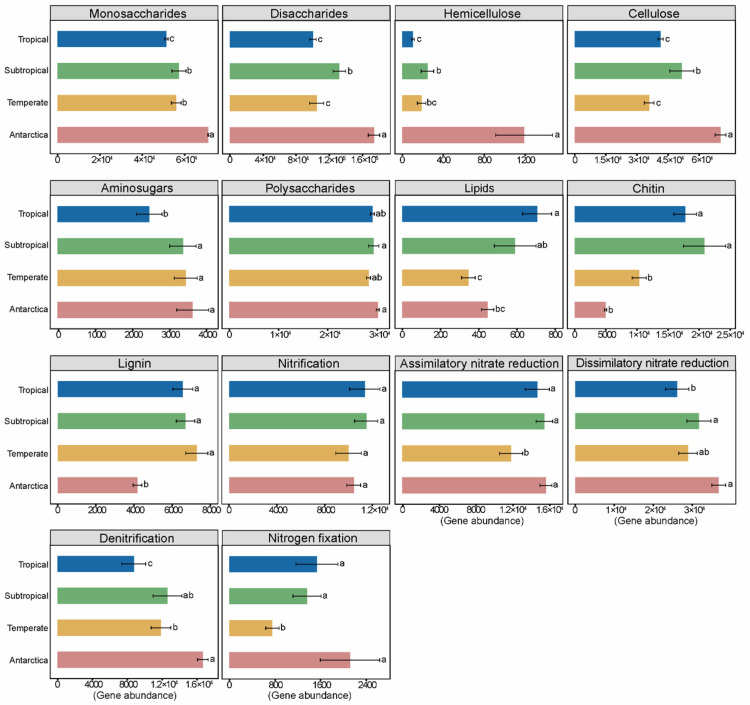
Function potentials of bacterial communities for studied sites. Functional potential differences related to labile C composition (monosaccharides, disaccharides, polysaccharides, hemicellulose, cellulose, and aminosugar), stable C composition (lipids, chitin, and lignin), and N cycling (assimilatory nitrate reduction, dissimilatory nitrate reduction, denitrification, nitrogen fixation, and nitrification) (mean ± SE). Different lowercase letters represent significant differences at 0.05 level.

Our results showed that genes involved in labile C decomposition and N cycling were more abundant in copiotroph-dominated Antarctic bacteria, and genes involved in stable C decomposition were more abundant in oligotroph-dominated temperate bacteria. In detail, compared to Antarctic soils, forest-inhabiting bacteria exhibited a 2.72–91.13% lower abundance of labile C decomposition genes (hemicellulose, cellulose, monosaccharides, and disaccharides) (Figure 3). Conversely, the abundance of stable C decomposition genes (lipids, chitin, and lignin) was more enriched in the forest soils (Figure 3). For example, the gene abundances of chitin and lignin in temperate, subtropical, and tropical forest soils were 107.67–318.06%, and 56.99–74.35% higher than in Antarctic soils, respectively (Figure 3). In addition, the gene abundances of assimilatory nitrate reduction, dissimilatory nitrate reduction, denitrification, and nitrogen fixation were 1.04–24.27%, 13.73–29.02%, 24.39–47.48%, and 27.35–64.43% lower in temperate, subtropical, and tropical forest soils than in Antarctic soils, respectively (Figure 3). However, the gene abundance of nitrification in temperate soils was 4.15% lower than that in Antarctic soils, while in subtropical and tropical soils it was 10.09% and 8.95 % higher than that in Antarctic soils, respectively (Figure 3).

## 4. Discussion 

### 4.1. The Selection of Bacterial Life Strategies under Different Soil Conditions

**Figure 4:**
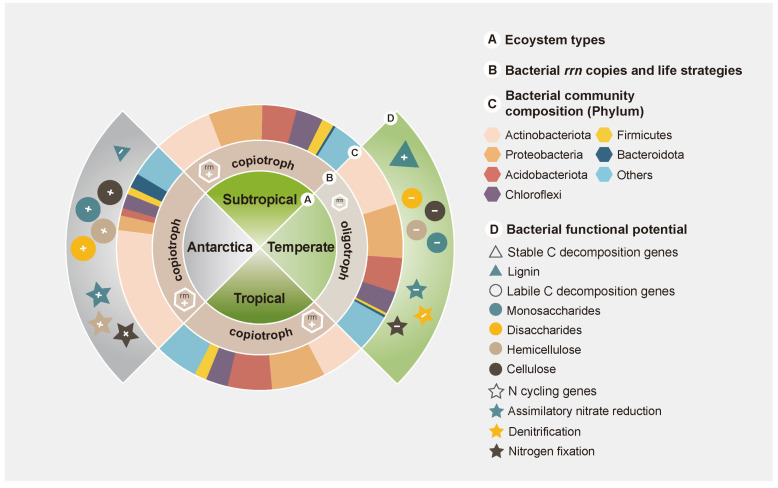
The concept graphic showing bacterial life strategies, community composition, and functional potential among four ecosystems. The graph consists of four rings, representing the ecosystem types (intermost), bacterial life strategies, bacterial community composition, and bacterial functional potential (outermost), respectively. The enrichment (+) and depletion (−) of *rrn* copies and functional potentials among four ecosystems are shown.

Inconsistent with our hypothesis, we found that *rrn* copies in temperate soils were significantly lower than those in Antarctic, subtropical, and tropical soils (*p* < 0.05; Figure 1 and Figure 4). The results indicated that bacterial communities in temperate soils tended to be oligotrophs, whereas those in subtropical, tropical, and Antarctic soils tended to be copiotrophs [10,30]. Such a phenomenon also contradicted the “distance decay theory”, in which bacteria in temperate forest ecosystems are likely to exhibit higher similarities with bacteria in Antarctic ecosystems compared to subtropical and tropical forest ecosystems [31,32,33]. 

The underlying mechanism that results in the inconsistency between temperate and Antarctic bacteria may involve two aspects. On the one hand, this phenomenon can mainly be attributed to soil C availability, since copiotrophic bacteria typically flourish in soil conditions rich in labile C with high C availability, while oligotrophic bacteria are more efficient with stable C with lower C availability [34,35]. Cui et al. [36] reported that copiotrophs proliferate following labile C inputs. Yang et al. [6] and Hu et al. [1] showed that increased soil C stability promoted a bacterial shift from copiotrophs to oligotrophs. Our results showed that soil C/N was significantly lower in Antarctic, subtropical, and tropical soils than in temperate soils and had a negative correlation with *rrn* copies (Table 1 and Table S2), indicating that higher C availability in Antarctic, subtropical, and tropical soils provided a more suitable site for the growth of copiotrophic bacteria. In contrast, higher soil C/N (lower C availability) in temperate soils suggests that substrate availability in temperate soil is more conducive to the proliferation of oligotrophs [37,38]. 

On the other hand, soil N availability may contribute to variations in bacterial life strategies [39]. Previous studies have reported that oligotrophic bacteria are more favorable under conditions of low N availability because oligotrophs can acquire N by decomposing soil organic matter (SOM) [14,40]. Temperate soils contain more SOM than Antarctic soils [37]; hence, the prevalence of oligotrophs was the underlying mechanism in temperate soils. Unlike bacteria in temperate soils, bacteria in Antarctic soil were dominated by copiotrophs, which may be associated with anoxic soil conditions, as well as large amounts of cellular exudates and dead microbial material from the labile C fraction in Antarctic soils [38,41]. Anoxic soil conditions can greatly limit SOM mineralization [36]. On another aspect, substances related to microbial turnover (e.g., dead microbial material) are an important component of microbial necromass [42]. Bacteria in Antarctic soils can utilize microbial necromass as an important N resource [43], which is a more efficient strategy for acquiring N than utilizing SOM [44].

### 4.2. Distinct Soil Conditions Shape Different Bacterial Structure and Functions: Based on Copiotrophs and Oligotrophs

Antarctic soil was inhabited by a large number of copiotrophs, such as Actinobacteriota and Bacteroidota (Figure 2, Figure 4 and Figure S3). Conversely, bacteria in temperate soils were mainly enriched in oligotrophs, such as Gammaproteobacteria, Acidobacteriota, and Chloroflexi (Figure 2, Figure 4 and Figure S3). Actinobacteriota are regarded as copiotrophs, which can use different C sources [45]. Bacteroidetes are also typical copiotrophs, inhabiting Antarctic soil conditions with high C availability (low soil C/N ratio) and can preferentially decompose labile carbon [4,16]. In contrast to copiotrophic bacteria, Acidobacteriota and Chloroflexi thrive in temperate soils with low resource availability and are thus considered oligotrophs [14,16]. Our results showed a significant positive correlation between NH_4_^+^-N and the relative abundance of Acidobacteriota and Chloroflexi (Table S3), further demonstrating that the dominant life strategy is largely influenced by resource availability [46].

In addition, our results revealed that oligotrophic and copiotrophic bacteria exhibited varying functional potentials for C/N cycling in temperate and Antarctic soils (Figure 3). Firstly, different capacities for soil C utilization by oligotrophs and copiotrophs could contribute to variations in labile C and stable C decomposition gene abundance [14,19]. Previous studies have reported that, compared to oligotrophic bacteria, copiotrophic bacteria are more adept at using labile C and have a higher abundance of labile C genes [14,34]. This is in line with our finding that genes involved in labile C (hemicellulose, cellulose, monosaccharides, and disaccharides) decomposition were more abundant in Antarctic soils, whereas genes involved in stable C (lignin) decomposition were more abundant in temperate soils (Figure 3 and Figure 4). Secondly, the lower N cycling (nitrogen fixation, assimilatory nitrate reduction, and denitrification) gene abundance of oligotrophs in temperate soils may be related to SOM utilization (Figure 3 and Figure 4). Liang et al. [18] found that the relative abundance of N cycling genes was lower in oligotrophs than in copiotrophs. This is because oligotrophs have the ability to acquire N by decomposing soil organic matter (SOM) [39]. Meanwhile, dominant copiotrophs in Antarctic soils chose to enhance their N fixation functional potential to acquire nitrogen [18,39,47]. Our results revealed that the abundance of nitrogen fixation and assimilatory nitrate reduction was positively correlated with *rrn* copies (Table S2), which further indicated that bacterial communities with contrasting life strategies had different N cycling potentials.

## 5. Conclusions

In the present study, we built a framework linking soil resource conditions, life strategies, bacterial community composition, and functional potential. We found that the bacterial communities in Antarctic, subtropical, and tropical soils were dominated by copiotrophs, whereas those in temperate soils were dominated by oligotrophs. Further analysis revealed that the bacterial community structure and functional potential showed different patterns between oligotrophic and copiotrophic bacteria. Overall, variations in soil conditions (e.g., soil substrate and climate) can affect bacterial life strategies through bacterial structure and functional potential. Our study systematically revealed differences in bacterial life strategies between Antarctic and forest ecosystems. We highlight that the differences in C and N cycling capabilities between copiotrophic and oligotrophic bacteria drive the carbon and nitrogen cycles in ecosystems, especially in the Antarctic ecosystem, where microbial processes are more sensitive than in other ecosystems. And future studies should focus on Antarctica, which is essential for better understanding of global biogeochemical processes.

## Figures and Tables

**Table 1:** Soil nutrients among four ecosystems (mean ± SE).

	Antarctica	Temperate	Subtropical	Tropical
pH	7.98 ± 0.31 a	6.47 ± 0.49 b	6.11 ± 0.21 b	5.69 ± 0.28 b
SOC (g/kg)	2.93 ± 0.12 c	56.46 ± 11.11 a	39.20 ± 4.70 b	27.06 ± 3.01 bc
MBC (mg/kg)	97.84 ± 0.56 b	522.04 ± 101.12 a	273.28 ± 33.61 b	254.69 ± 25.14 b
NH_4_^+^-N (mg/kg)	3.89 ± 1.53 b	25.80 ± 3.11 a	29.19 ± 3.28 a	24.25 ± 0.34 a
NO_3_^−^-N (mg/kg)	1.70 ± 0.71 c	12.50 ± 3.17 ab	16.45 ± 2.58 a	10.36 ± 1.70 b
C/N	13.67 ± 0.17 b	28.45 ± 3.35 a	15.04 ± 1.40 b	13.25 ± 0.82 b

Notes: SOC—soil organic carbon; MBC—soil microbial biomass C; and C/N—the ratio of soil organic carbon to total nitrogen. Different letters indicate the significant level (*p* < 0.05).

## Data Availability

All the sequencing data have been deposited on the National Center for Biotechnology Information website (SRP504188). The data and scripts used are saved in GitHub https://github.com/zhanglujie1234/data-and-code.git (accessed on 15 August 2024).

## Supplementary Materials

The following supporting information can be downloaded at: https://www.mdpi.com/article/10.3390/microorganisms12081689/s1, Figure S1: Distribution of sampling sites along forest ecosystems in China and Antarctic ecosystem.; Figure S2: Diversity of bacterial communites among four ecosystems. (a) Shannon’s diversity index; (b) observed species richness; (c) Non-metric multidimensional scaling (NMDS) plot based on Bray-Curtis dissimilarities among four ecosystems; Figure S3: Relative abundance of soil bacterial communities at the phylum level among four ecosystems; Figure S4: LEfSe analysis of bacterial abundance among four ecosystems. Indicator bacteria with LDA scores of 3 or greater in bacterial communities among four ecosystems; Table S1: Backgroud information at study sites; Table S2: Spearman analysis between *rrn* copies and (a) environmental conditions, (b) soil nutrient, (c) bacterial functional potential. *, *p* < 0.05; **, *p* < 0.01; Table S3: Spearman analysis betweem soil nutrient and bactetial community at the phylum level. *, *p* < 0.05; **, *p* < 0.01. References [48,49,50,51,52] are listed in the Supplementary Material.
