# Supplementary material for: Antarctic Soils Select Copiotroph-Dominated Bacteria
Source: Microorganisms. 2024 Aug 16;12(8):1689. doi: 10.3390/microorganisms12081689 (PMC11357078; doi:10.3390/microorganisms12081689)
Supplement: Supplementary file 1 [file microorganisms-12-01689-s001.zip › microorganisms-3167333-supplementary.pdf]

Supplementary Methods

Antarctic soils select copiotroph-dominated bacteria

Figure S1 Distribution of sampling sites along forest ecosystems in China and Antarctic ecosystem.

Figure S2 Diversity of bacterial communites among four ecosystems. (a) Shannon's diversity index; (b) observed species richness; (c) Non-metric multidimensional scaling (NMDS) plot based on Bray-Curtis dissimilarities among four ecosystems.

Figure S3 Relative abundance of soil bacterial communities at the phylum level among four ecosystems.

Figure S4 LEfSe analysis of bacterial abundance among four ecosystems. Indicator bacteria with LDA scores of 3 or greater in bacterial communities among four ecosystems.

Table S1 Backgroud information at study sites.

Table S2 Spearman analysis between *rrn* copies and (a) environmental conditions, (b) soil nutrient, (c) bacterial functional potential. *, $p < 0.05$; **, $p < 0.01$.

Table S3 Spearman analysis betweem soil nutrient and bactetial community at the phylum level. *, $p < 0.05$; **, $p < 0.01$.

Supplementary Information

DNA extraction, PCR amplification, and data processing

Following the manufacturer's instructions, the total genomic DNA samples were extracted using the E.Z.N.A.® soil DNA Kit (Omega Bio-tek, Norcross, GA, U.S.), and stored at -20°C prior to further analysis. The quality and concentration of DNA were determined by 1.0% agarose gelelectrophoresis and a NanoDrop2000 spectrophotometer (Thermo Scientific, United States).

The V3–V4 region of 16S rRNA was performed using the forward primer 338F (5'-ACTCCTACGGGAGGCAGCA-3') and the reverse primer 806R (5'-GGACTACHVGGGTWTCTAAT-3'). Sample-specific 7-bp barcodes were incorporated into the primers for multiplex sequencing. The PCR reaction mixture including 5 μl of buffer (5×), 0.25 μl of Fast pfu DNA Polymerase (5U/μl), 2 μl (2.5 mM) of dNTPs, 1 μl (10 uM) of each Forward and Reverse primer, 1 μl of DNA Template, and 14.75 μl of ddH_2O. Thermal cycling consisted of initial denaturation at 98°C for 5 min, followed by 25 cycles consisting of denaturation at 98°C for 30 s, annealing at 53°C for 30 s, and extension at 72°C for 45 s, with a final extension of 5 min at 72°C. PCR amplicons were purified with Vazyme VAHTSTM DNA Clean Beads (Vazyme, Nanjing, China) and quantified using the Quant-iT PicoGreen dsDNA Assay Kit (Invitrogen, Carlsbad, CA, USA). After the individual quantification step, amplicons were pooled in equal amounts, and pair-end 2×250 bp sequencing was performed using the Illlumina NovaSeq platform with NovaSeq 6000 SP Reagent Kit

(500 cycles) at Shanghai Personal Biotechnology Co., Ltd (Shanghai, China).

Raw FASTQ files were de-multiplexed using an in-house perl script, and then quality-filtered by fastp version 0.19.6 (Chen et al., 2018) and merged by FLASH version 1.2.7 (Magoč and Salzberg, 2011) with the following criteria: (1) The reads were truncated at any site receiving an average quality score of <20 over a 50 bp sliding window, and the truncated reads shorter than 50 bp were discarded, reads containing ambiguous characters were also discarded; (2) only overlapping sequences longer than 10 bp were assembled according to their overlapped sequence. The maximum mismatch ratio of overlap region is 0.2. Reads that could not be assembled were discarded; (3) samples were distinguished according to the barcode and primers, and the sequence direction was adjusted, exact barcode matching, 2 nucleotide mismatch in primer matching.

Then the optimized sequences were clustered into operational taxonomic units (OTUs) using UPARSE 7.1 (Edgar, 2013; E and M, 1994) with 97% sequence similarity level. The most abundant sequence for each OTU was selected as a representative sequence. The OTUs assigned to spike-in sequences were filtered out and reads were counted. A Standard curves (based on read counts versus spike-in DNA copy number) for each sample were generated, the quantitative abundance of each OTU in a sample was determined. The taxonomy of each OTU representative sequence was analyzed by RDP Classifier version 2.2 (Wang et al., 2007) against the 16S rRNA gene database (eg. Silva v138) using confidence threshold of 0.7, and then adjusted on the basis of the

estimated rRNA operon copy number according to the rrnDB database (Stoddard et al., 2015).

Figure S2

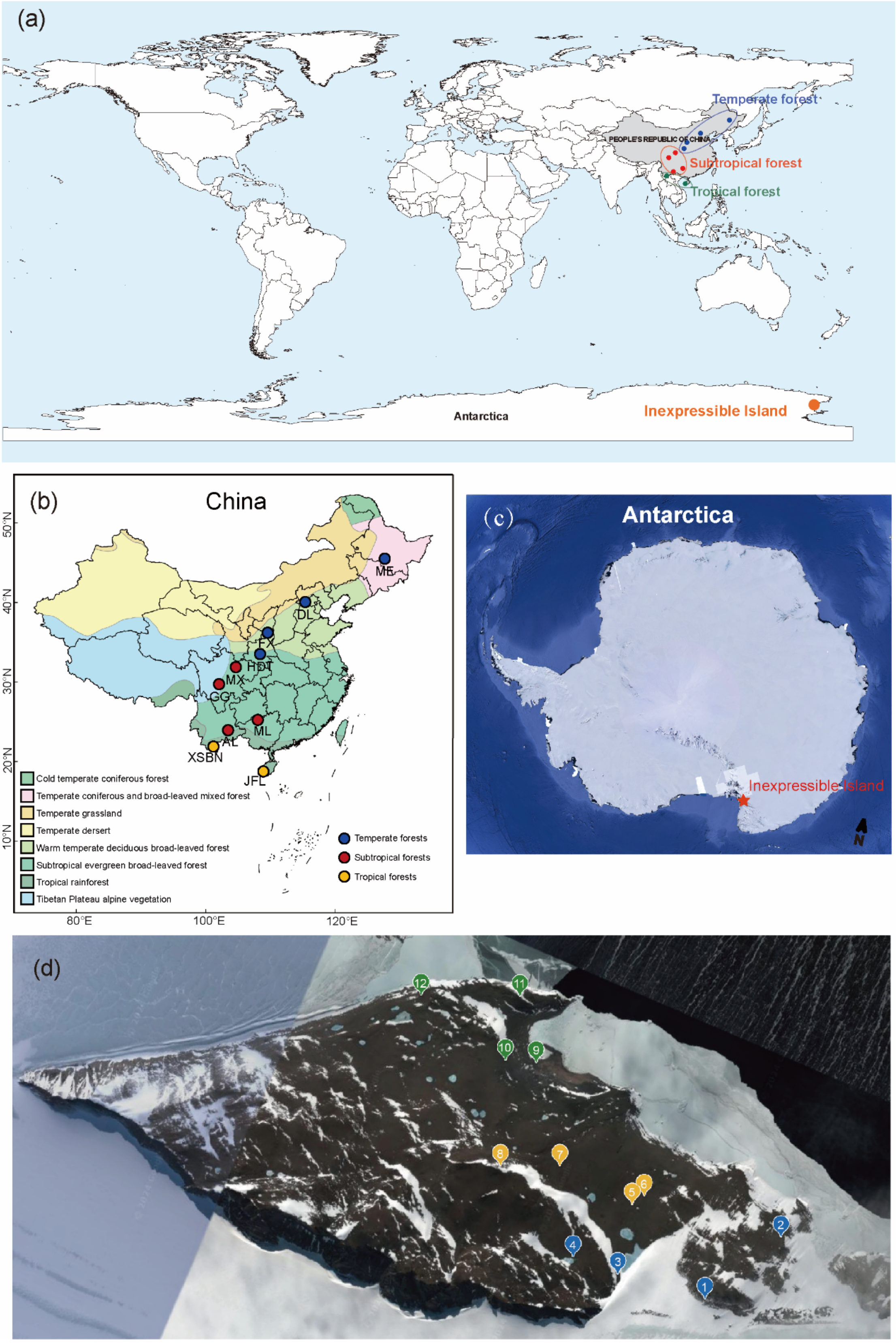

(a)
Temperate forest
PEOPLE'S REPUBLIC OF CHINA
Subtropical forest
Tropical forest
Antarctica
Inexpressible Island
(b)
China
ME
DL
FX
HDT
MX
GG
ML
AL
XSBN
JFL
50°N
40°N
30°N
20°N
10°N
80°E
100°E
120°E
Cold temperate coniferous forest
Temperate coniferous and broad-leaved mixed forest
Temperate grassland
Temperate dersert
Warm temperate deciduous broad-leaved forest
Subtropical evergreen broad-leaved forest
Tropical rainforest
Tibetan Plateau alpine vegetation
Temperate forests
Subtropical forests
Tropical forests
(c)
Antarctica
Inexpressible Island
N
(d)
1
2
3
4
5
6
7
8
9
10
11
12

Figure S2

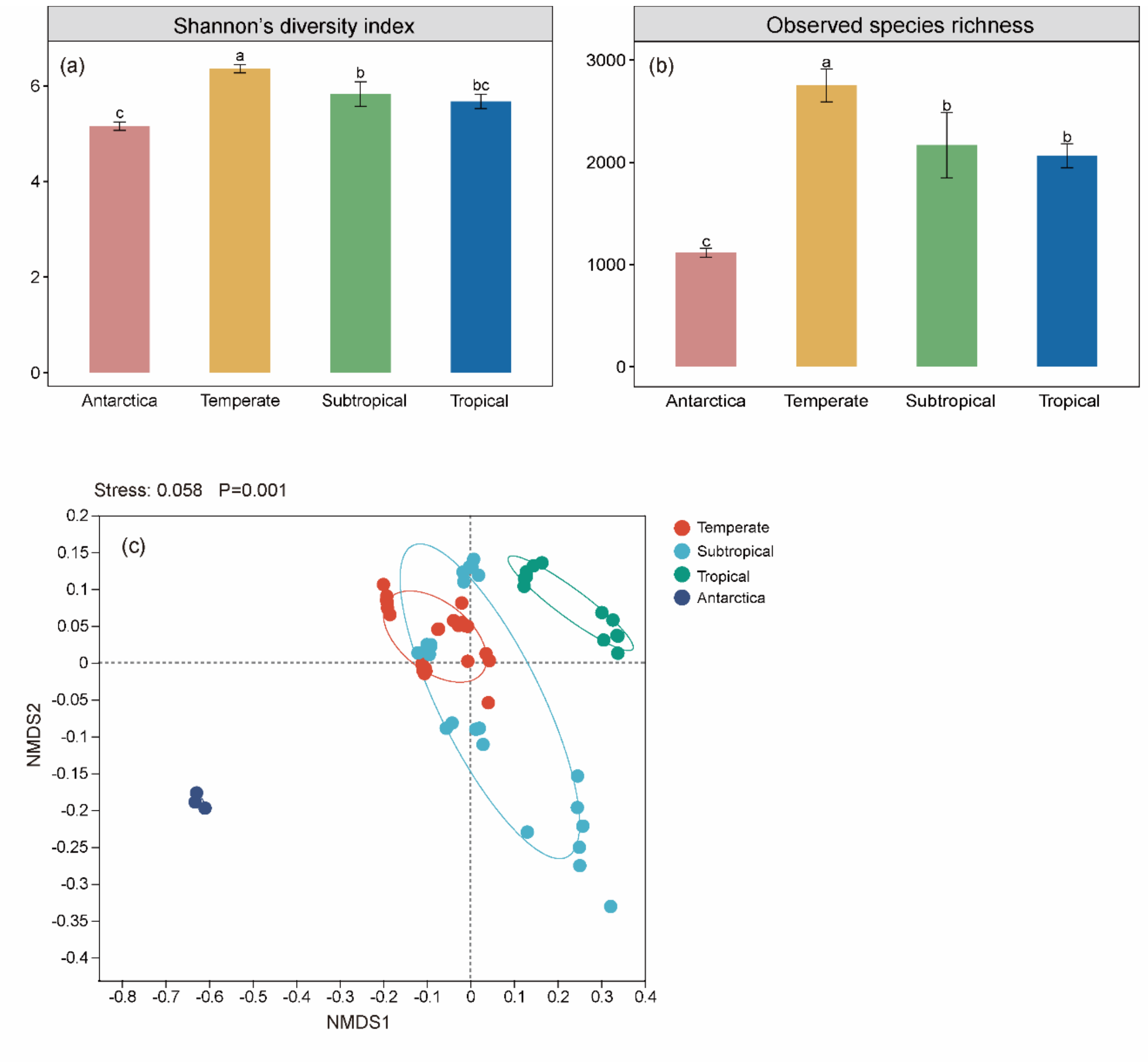

Shannon's diversity index
(a)
0
2
4
6
c
a
b
bc
Antarctica
Temperate
Subtropical
Tropical
Observed species richness
(b)
0
1000
2000
3000
c
a
b
b
Antarctica
Temperate
Subtropical
Tropical
Stress: 0.058 P=0.001
(c)
NMDS2
NMDS1
0.2
0.15
0.1
0.05
0
-0.05
-0.1
-0.15
-0.2
-0.25
-0.3
-0.35
-0.4
-0.8
-0.7
-0.6
-0.5
-0.4
-0.3
-0.2
-0.1
0
0.1
0.2
0.3
0.4
Temperate
Subtropical
Tropical
Antarctica

Figure S3

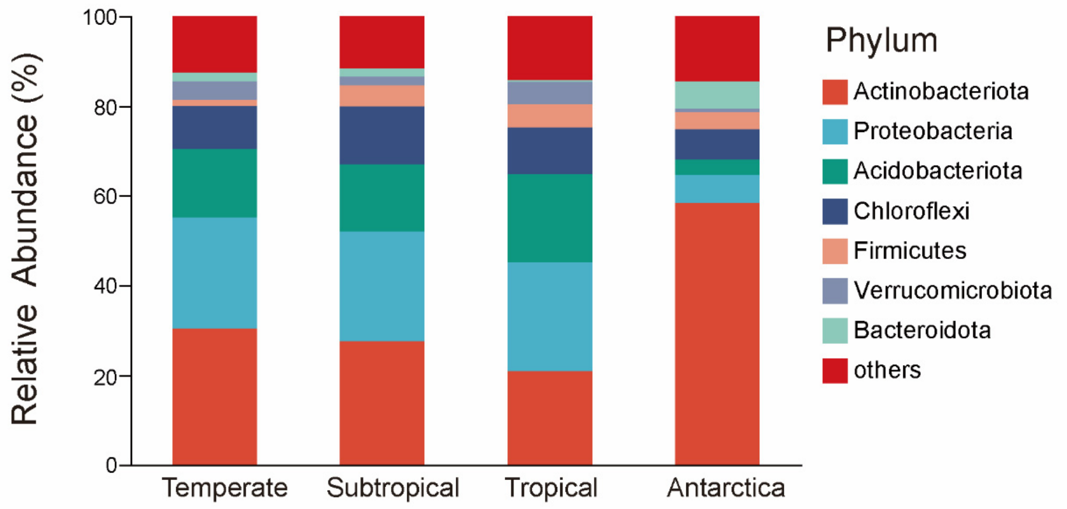

Relative Abundance (%)
100
80
60
40
20
0
Temperate
Subtropical
Tropical
Antarctica
Phylum
Actinobacteriota
Proteobacteria
Acidobacteriota
Chloroflexi
Firmicutes
Verrucomicrobiota
Bacteroidota
others

Figure S4

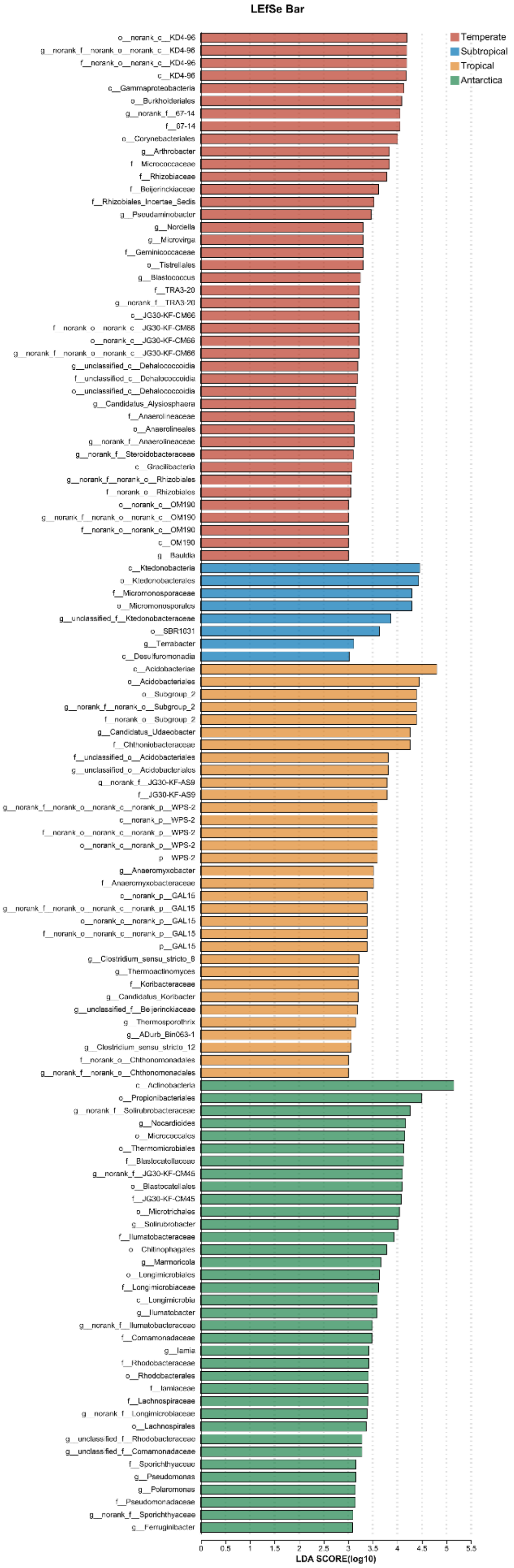

LEfSe Bar
Temperate
Subtropical
Tropical
Antarctica
o__norank_c__KD4-96
g__norank_f__norank_o__norank_c__KD4-96
f__norank_o__norank_c__KD4-96
c__KD4-96
c__Gammaproteobacteria
o__Burkholderiales
g__norank_f__67-14
f__67-14
o__Corynebacteriales
g__Arthrobacter
f__Micrococcaceae
f__Rhizobiaceae
f__Beijerinckiaceae
f__Rhizobiales_Incertae_Sedis
g__Pseudaminobacter
g__Nordella
g__Microvirga
f__Geminicoccaceae
o__Tistrellales
g__Blastococcus
f__TRA3-20
g__norank_f__TRA3-20
c__JG30-KF-CM66
f__norank_o__norank_c__JG30-KF-CM66
o__norank_c__JG30-KF-CM66
g__norank_f__norank_o__norank_c__JG30-KF-CM66
g__unclassified_c__Dehalococcoidia
f__unclassified_c__Dehalococcoidia
o__unclassified_c__Dehalococcoidia
g__Candidatus_Alysiosphaera
f__Anaerolineaceae
o__Anaerolineales
g__norank_f__Anaerolineaceae
g__norank_f__Steroidobacteraceae
c__Gracilibacteria
g__norank_f__norank_o__Rhizobiales
f__norank_o__Rhizobiales
o__norank_c__OM190
g__norank_f__norank_o__norank_c__OM190
f__norank_o__norank_c__OM190
c__OM190
g__Bauldia
c__Ktedonobacteria
o__Ktedonobacterales
f__Micromonosporaceae
o__Micromonosporales
g__unclassified_f__Ktedonobacteraceae
o__SBR1031
g__Terrabacter
c__Desulfuromonadia
c__Acidobacteriae
o__Acidobacteriales
o__Subgroup_2
g__norank_f__norank_o__Subgroup_2
f__norank_o__Subgroup_2
g__Candidatus_Udaeobacter
f__Chthoniobacteraceae
f__unclassified_o__Acidobacteriales
g__unclassified_o__Acidobacteriales
g__norank_f__JG30-KF-AS9
f__JG30-KF-AS9
g__norank_f__norank_o__norank_c__norank_p__WPS-2
c__norank_p__WPS-2
f__norank_o__norank_c__norank_p__WPS-2
o__norank_c__norank_p__WPS-2
p__WPS-2
g__Anaeromyxobacter
f__Anaeromyxobacteraceae
c__norank_p__GAL15
g__norank_f__norank_o__norank_c__norank_p__GAL15
o__norank_c__norank_p__GAL15
f__norank_o__norank_c__norank_p__GAL15
p__GAL15
g__Clostridium_sensu_stricto_8
g__Thermoactinomyces
f__Koribacteraceae
g__Candidatus_Koribacter
g__unclassified_f__Beijerinckiaceae
g__Thermosporothrix
g__ADurb_Bin063-1
g__Clostridium_sensu_stricto_12
f__norank_o__Chthonomonadales
g__norank_f__norank_o__Chthonomonadales
c__Actinobacteria
o__Propionibacteriales
g__norank_f__Solirubrobacteraceae
g__Nocardioides
o__Micrococcales
o__Thermomicrobiales
f__Blastocatellaceae
g__norank_f__JG30-KF-CM45
o__Blastocatellales
f__JG30-KF-CM45
o__Microtrichales
g__Solirubrobacter
f__Ilumatobacteraceae
o__Chitinophagales
g__Marmoricola
o__Longimicrobiales
f__Longimicrobiaceae
c__Longimicrobia
g__Ilumatobacter
g__norank_f__Ilumatobacteraceae
f__Comamonadaceae
g__Iamia
f__Rhodobacteraceae
o__Rhodobacterales
f__Iamiaceae
f__Lachnospiraceae
g__norank_f__Longimicrobiaceae
o__Lachnospirales
g__unclassified_f__Rhodobacteraceae
g__unclassified_f__Comamonadaceae
f__Sporichthyaceae
g__Pseudomonas
g__Polaromonas
f__Pseudomonadaceae
g__norank_f__Sporichthyaceae
g__Ferruginibacter
0
0.5
1
1.5
2
2.5
3
3.5
4
4.5
5
5.5
LDA SCORE(log10)

Table S1

Ecosysytem type	Sample site	Elevation (m)	Latitude (°)	Longtitude (°)	MAP (mm)	MAT (°C)
Temperate forest	ME	600	45.41 N	127.71E	629	3.10
	DL	1300	39.97 N	115.43E	650	5.00
	FX	1035	36.11 N	109.63 E	580	7.40
	HDT	1526	33.43 N	108.45E	1023	9.00
Subtropical forest	MX	1625	31.79 N	104.71E	486	11.00
	GG	1726	29.65 N	102.11 E	1000	13.00
	ML	652	25.13 N	108.00 E	1529	19.38
	AL	2300	23.87 N	103.51 E	1086	18.30
Tropical forest	XSBN	560	21.83 N	101.20 E	1500	21.60
	JFL	820	18.71 N	108.91 E	2266	23.15
Antarctica	Inexpressible Island		74.93 S	163.66 E	100-200	-18.5
			74.93 S	163.70 E		
			74.92 S	163.65 E		
			74.91 S	163.65 E		
			74.92 S	163.68 E		
			74.92 S	163.69 E		
			74.90 S	163.69 E		
			74.90 S	163.68 E		
			74.90 S	163.73 E		
			74.89 S	163.72 E		
			74.89 S	163.75 E		
			74.88 S	163.74 E		

Maoer Mountain (ME), Dongling Mountain (DL), Fuxian (FX), Huoditang (HDT), Maoxian (MX), Gongga Mountain (GG), Ailao Mountain (AL), Mulun (ML), Xishuangbanna (XSBN), Jianfengling (JFL). MAT, mean annual temperature; MAP, mean annual precipitation

Table S2

(a)

	pH	MAP	MAT
rrn	0.164	0.373	0.527

(b)

	SOC	NH_4^+-N	NO_3^--N	MBC	C/N
rrn	-0.427	-0.436	-0.427	-0.364	-0.827**

(c)

	Aminosugars	Cellulose	Chitin	Disaccharides	Hemicellulose
rrn	-0.147	0.347**	0.308*	0.383**	-0.024

	lignin	Lipids	Monosaccharides	Polysaccharides
rrn	-0.370**	0.538**	-0.161	0.392**

	Assimilatory nitrate reduction	Denitrification	Dissimilatory nitrate reduction	Nitrification	Nitrogen fixation
rrn	0.357**	-0.152	-0.075	0.237	0.295*

Table S3

	SOC	NH_4^+-N	NO_3^--N	MBC	C/N
Acidobacteriota	-0.036	0.718*	-0.1	-0.091	0.209
Actinobacteriota	0.045	-0.473	0.145	0.173	0.364
Bacteroidota	0.136	-0.455	-0.118	0.145	-0.036
Chloroflexi	-0.1	0.736**	0.2	-0.182	0.355
Cyanobacteria	-0.636*	-0.255	-0.464	-0.673*	-0.264
Desulfobacterota	0.264	0.173	0.045	0.282	-0.345
Entotheonellaeota	0.733*	0.105	0.328	0.866**	0.323
Firmicutes	-0.191	-0.445	-0.445	-0.009	-0.618*
GAL15	-0.041	0.351	0.064	0.214	0.05
Gemmatimonadota	0.073	-0.309	-0.327	0.318	0.264
Latescibacterota	0.609*	0.091	-0.1	0.682*	0.009
Myxococcota	0.582	-0.1	0.127	0.527	0.136
NB1-j	0.627*	0.3	0.173	0.491	-0.055
Proteobacteria	0.018	0.345	0.364	-0.1	-0.127
RCP2-54	-0.236	0.736**	-0.145	-0.236	0.155
Verrucomicrobiota	0.636*	0.136	0.327	0.627*	0.464
WPS-2	-0.227	0.345	0.027	-0.4	-0.091
WS2	0.578	-0.018	0.275	0.633*	0.477

Supporting reference

Chen S, Zhou Y, Chen Y, Gu J. 2018. fastp: an ultra-fast all-in-one FASTQ preprocessor. Bioinformatics. 34: i884-i890.

Stackebrandt, E, Goebel, B M. 1994. Taxonomic Note: A Place for DNA-DNA Reassociation and 16S rRNA Sequence Analysis in the Present Species Definition in Bacteriology. Int.j.syst.bacteriol. 44: 846-849.

Edgar R C. 2013. UPARSE: highly accurate OTU sequences from microbial amplicon reads. Nature Methods. 10: 996-998.

Magoč T, Salzberg S L. 2011. FLASH: fast length adjustment of short reads to improve genome assemblies. Bioinformatics. 27: 2957-2963.

Stoddard S F, Smith B J, Hein R, Roller B R K, Schmidt T M. 2015. rrnDB: improved tools for interpreting rRNA gene abundance in bacteria and archaea and a new foundation for future development. Nucleic Acids Research. 43: D593-D598.

Wang Q, Garrity G M, Tiedje J M, Cole J R. 2007. Naïve Bayesian Classifier for Rapid Assignment of rRNA Sequences into the New Bacterial Taxonomy. Applied and Environmental Microbiology. 73: 5261-5267.
